# Demography, maternal health and the epidemiology of malaria and other major infectious diseases in the rural department Tsamba-Magotsi, Ngounie Province, in central African Gabon

**DOI:** 10.1186/s12889-017-4045-x

**Published:** 2017-01-28

**Authors:** R. Zoleko Manego, G. Mombo-Ngoma, M. Witte, J. Held, M. Gmeiner, T. Gebru, B. Tazemda, J. Mischlinger, M. Groger, B. Lell, A. A. Adegnika, S. T. Agnandji, P. G. Kremsner, B. Mordmüller, M. Ramharter, P. B. Matsiegui

**Affiliations:** 1 Centre de Recherches Médicales de Lambaréné , Lambaréné, Gabon; 2 Institut für Tropenmedizin, University of Tübingen, 72074 Tübingen, Germany; 3 Département de Parasitologie-Mycologie, Université des Sciences de La Santé, Libreville, Gabon; 4 Deutsches Zentrum für Infektionsforschung (DZIF), Standort, Tübingen, Germany; 5 Department of Medicine I, Division of Infectious Diseases and Tropical Medicine, Medical University of Vienna, Vienna, Austria; 6 Centre de Recherches Médicales de la Ngounié, Fougamou, Gabon

**Keywords:** Malaria, Filariasis, Urinary schistosomiasis, Pregnancy, Maternal health, Gabon, Tsamba-Magotsi

## Abstract

**Background:**

Sub-Saharan Africa is undergoing an epidemiological transition from a predominance of infectious diseases to non-communicable and lifestyle related conditions. However, the pace of this transition and the pattern of disease epidemiology are uneven between affluent urban and rural poor populations. To address this question for a remote rural region located in the central African rainforest region of Gabon, this study was conducted to assess reasons for health care attendance and to characterize the epidemiology of malaria and other major infectious diseases for the department of Tsamba Magotsi.

**Methods:**

Major causes for health care attendance were collected from local hospital records. Cross sectional population based surveys were performed for the assessment of local malaria epidemiology. Pregnant women attending antenatal care services were surveyed as a sentinel population for the characterization of chronic viral and parasitic infections in the community.

**Results:**

Infectious diseases were responsible for 71% (7469) of a total of 10,580 consultations at the formal health care sector in 2010. Overall, malaria – defined by clinical syndrome – remained the most frequent cause for health care attendance. A cross sectional malaria survey in 840 asymptomatic individuals residing in Tsamba Magotsi resulted in a *Plasmodium spp.* infection prevalence of 37%. The infection rate in 2–10 year old asymptomatic children – a standard measure for malaria endemicity – was 46% (100 of 217) with *P. falciparum* as predominant species (79%). Infection with other plasmodial species (*P. ovale* and *P. malariae*) presented most commonly as coinfections (23.2%). Prevalence of HIV, HBV, and syphilis were 6.2, 7.3, and 2.5%, respectively, in cross-sectional assessments of antenatal care visits of pregnant women. Urogenital schistosomiasis and the filarial pathogens *Loa loa* and *Mansonella perstans* are highly prevalent chronic parasitic infections affecting the local population.

**Conclusions:**

Despite major improvements in the accessibility of Tsamba Magotsi over the past decade the epidemiological transition does not appear to have majorly changed on the spectrum of diseases in this rural Gabonese population. The high prevalence of *Plasmodium* infection indicates a high burden of malaria related morbidity. Infectious diseases remain one of the most important health issues and further research activities in the field of tropical medicine and infectious diseases could help improve health care for the local population.

**Electronic supplementary material:**

The online version of this article (doi:10.1186/s12889-017-4045-x) contains supplementary material, which is available to authorized users.

## Background

Sub-Saharan Africa is at the same time in an epidemiological and a demographic transition. Whereas infectious diseases have been the leading cause of morbidity and mortality in the past, the prevalence and impact of non-communicable diseases are rising rapidly in African populations [1]. This is illustrated by the fact that adults under the age of 70 years are today at a higher risk for death from a non-communicable disease in sub-Saharan Africa than adults of the same age in high income countries [2].

This epidemiological transition and concurrent changes in fertility and family size are however occurring at an uneven pace between urban and rural African populations [1]. Urban populations in sub-Saharan Africa are of increasing demographic as well as economic importance. This development is paralleled by rural depopulation and rapid growth of African cities [3]. However, despite these economic and demographic changes rural populations residing in remote regions must not be neglected. Up to date epidemiological surveys are needed to identify changing priorities for human health. Adequate information about the health of rural populations is even more essential in countries with highly advanced urbanization such as Gabon, where 86% of the population is living in urban and peri-urban regions [4].

The department of Tsamba-Magotsi is located in the landlocked province of Ngounié in southern-central Gabon. To date no reliable data on the epidemiology and spectrum of human diseases have been published for this rural region. The establishment of the medical research centre "Centre de Recherches Médicales de la Ngounié" (CRMN) as an independent research institution provided a unique opportunity for the description of the epidemiology of major diseases prevalent in this region. Here, reasons of health care attendance and the epidemiology of major infectious diseases of the population residing in this remote region of Gabon are described.

## Methods

### Study region and population

This study was conducted in the department of Tsamba-Magotsi between January 2008 and March 2016. Tsamba-Magotsi is a second-order administrative division of Ngounié province in southern-central Gabon [5]. It has a North–south and East–west extension of approximately 135 km and 75 km, respectively. The Waka National Park protecting over 1,000 km^2^ of rain forest in the Chaillu Massif is almost entirely located in this department.

The small town of Fougamou serves as administrative centre for this department and its surrounding rural villages. The region is located at approximately 230 meters above sea level and is covered by tropical rainforest. Climate is equatorial and there are two rainy seasons occurring from September to November and from February to May [6]. Main commercial activities of the local population include subsistence farming, game hunting and fishing besides few employments in the governmental administrative sector. This region is the traditional heartland of the Ghisir and the mitsogo ethnic groups and their important cultural and historic heritage.

The Ngounié Medical Research Centre in Fougamou was founded in 2007 as a satellite site of the Centre de Recherches Médicales de Lambaréné (CERMEL) and later became an independent research institution [7]. Applied medical and epidemiological research is the primary focus of this research centre and it has successfully participated as study site in international multi-centre trials [8, 9]. Other major activities include assistance for the national antenatal care program and provision of laboratory diagnostic services for the governmental Centre Médical de Fougamou, which is the only formal health care facility in this department besides one rural health center in the Pygmy and the Mitsogo communities of Ikobey and 16 dispensaries located in the larger villages.

### Study design and procedures

Data for this epidemiological review stem from several independent surveys (Additional file 1: Table S1). Hospital records documenting the primary reason for attendance were assessed and demographic data about patients were collected from hospital registries. Data from antenatal care visits of pregnant women were obtained from antenatal clinic and the research centre’s registries including maternal age, obstetric history, gestational age, anthropometric measures, haemoglobin, HIV (VIKIA® HIV ½v Determine® or Uni-cold HIV), hepatitis B (Vikia® HBsAg), toxoplasmosis, rubella, and syphilis (Alere Determine™ Syphilis and TPHA 100) rapid testing, as well as malaria microscopy using the Lambaréné method [10] birth weight and pregnancy outcome. A cross sectional community survey evaluating the epidemiology of malaria was conducted between February and March 2016 determining the prevalence of *Plasmodium* infection by Giemsa stained thick blood smear microscopy. Urogenital schistosomiasis and filarial infections were assessed in pregnant women participating in a clinical trial for prevention of malaria during pregnancy conducted in Fougamou [11–13]. To assess urogenital schistosomiasis, urine filtration method was used. Microfilaria detection was performed either by thick or thin film or by leuco-concentration through (EDTA) venous blood (1 ml) [11].

### Data management and statistical analysis

Data were transcribed from source records to an electronic database. Consistence was checked by a semi-automated procedure and by manual review. Data were transferred to a commercial statistical software package (JMP 10.0, SAS, US) for further descriptive analysis.

## Results

### Demography and disease burden

The latest accurate demographic data for the department of Tsamba-Magotsi derive from a governmental census performed in the year 2003 [5]. The total population was estimated at 12,000 inhabitants with 4,400 residing in the town of Fougamou. Almost half of the population was younger than 19 years (48%) and there was a sex ratio of 0.9 (male/female). A recent governmental estimation for the population of Tsamba-Magotsi put the total population of Tsamba Magotsi at 14430 inhabitants and the population of Fougamou at 3745. In the year 2010 10,580 consultations of patients were performed at the governmental hospital or affiliated health posts and approximately 1,000 patients were hospitalized during this period. Infectious diseases were responsible for 71% of hospital consultations. Parasitologically confirmed and clinically suspected malaria accounted for 3,491 consultations (33%). Other common communicable diseases included infections of the gastrointestinal system (*n* = 1,646; 16%), the respiratory tract (*n* = 1,302; 12%), soft tissues (*n* = 142; 1%), and the urogenital tract (*n* = 99; 1%). Conditions of the musculo-skeletal system (*n* = 803; 8%), injury (*n* = 522; 5%), anaemia (*n* = 172; 2%), and cardiovascular disease (*n* = 92; 1%) were the most important causes for health care seeking for non-communicable diseases.

### Epidemiology of malaria and other relevant parasitic diseases

**Fig. 1:**
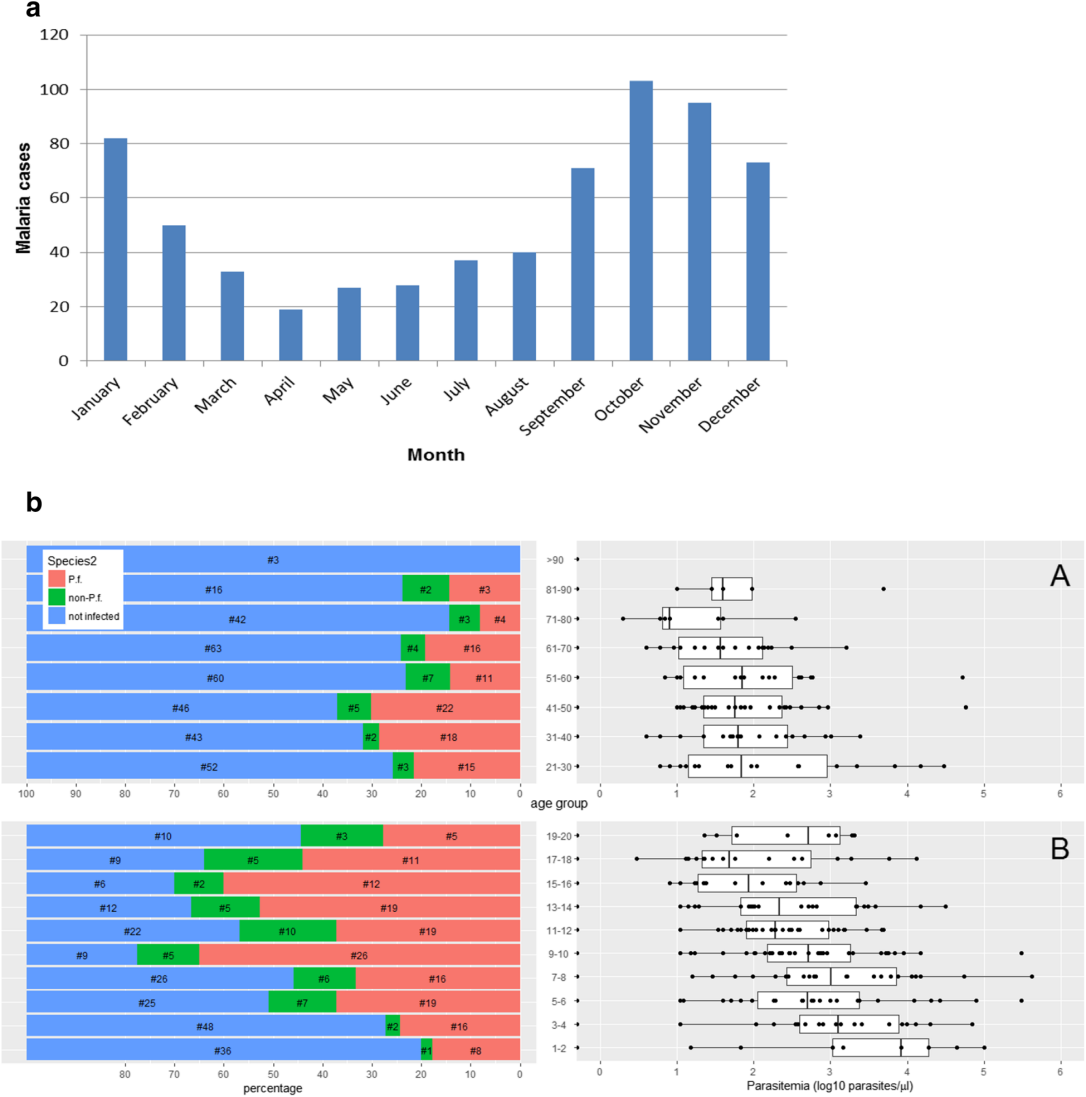
**a** Seasonal trends in parasitologically confirmed malaria cases at the centre medical Fougamou in the year 2008). **b** Proportion of *Plasmodium* infected individuals and parasitemia per age category in a cross sectional survey of asymptomatic individuals in February/March 2016. *a*: individual older than 20 years and *b*: individual below or equal to 20 years. Percentage of Plasmodium infected individuals (subdivided in *P. falciparum*, non-falciparum and non-infected individuals) depicted on left side. Number of individuals per age category indicated in Log 10 parasitemia (median, IQR and 95% whiskers) per μl is shown on right side)

Malaria transmission is perennial with a reduced transmission rate during the dry seasons and highest incidence during the rainy seasons. The distribution of parasitologically confirmed malaria cases is depicted exemplarily in Fig. 1a.

A cross-sectional community survey of individuals of all ages (ranging from 1–96 years) was performed in February-March 2016. A total of 840 participants were investigated by microscopy for *Plasmodium* infection and in 311 (37%) individuals the presence of parasites was confirmed. Out of these parasitaemic subjects, 72 (23.2%) were infected with other species than *P. falciparum*. Due to the low parasitemia and consequently unreliable microscopic distinction between the species of *P. malariae* and *P. ovale* the two species were only reported as non-*falciparum* species. The number of screened persons per age group and the percentage of *Plasmodium* positive individuals together with their median parasitaemia is depicted in Fig. 1b

Prevalence of Plasmodium infection was highest in children and adolescents between 5 and 20 years of age with infection rates reaching up to 77% (Fig. 1b). Prevalence in children aged 2–10 years is commonly used as standardized measure for malaria endemicity. In this age group 100 out of 217 (46%) individuals were infected.

Median parasite count in all individuals was 185 (IQR: 34–1020) parasites/μl. Children ≤ 10 years had 8.2-fold higher parasite counts (median 700 parasites/μl IQR: 185–5786) than older children and adults (median 86 parasites/μl IQR: 22–407). Children aged 2–10 years had a median parasitemia of 695 (IQR 182–4571) parasites/μl.

From August 2008 to February 2009, a total of 1,180 patients were evaluated for the presence of microfilaria by microscopy. The overall carriage rate for microfilaria was 15% (*n* = 171) with 7% (*n* = 86) *Loa loa*, 5% (*n* = 60) *Mansonella perstans* and 2% (*n* = 25) mixed infection. The proportion of microfilaria infection in patients attending health care in Fougamou increased with age. Microfilaria were detected respectively in 7% (47/682) of patients under 20 years, 23% (56/239) of 20 to 39 years old, 22% (37/165) of 40 to 60 years old and 27% (22/81) in patients above 60 years old. History of eye worm (10%, *n* = 25), pruritis (30%, *n* = 77) and Calabar swelling (22%, *n* = 56) were the most frequently observed clinical manifestations. About 11% (*n* = 126) of those complaining about these symptoms were positive for microfilaria while 4% (*n* = 45) of those without symptoms were thin smear positive for microfilaria.

### Maternal health and epidemiology of infectious diseases in pregnancy

**Fig. 2:**
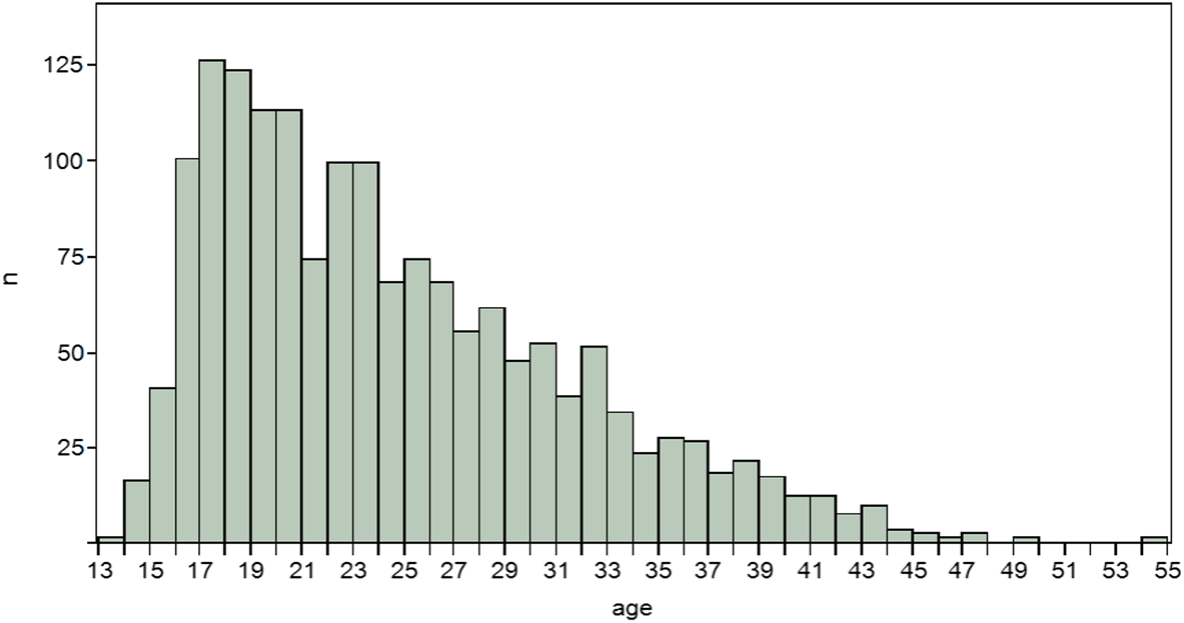
age distribution of pregnant women attended the centre medical de Fougamou 2008–2011

A total of 1,665 pregnant women attended Fougamou’s maternity ward as part of antenatal care visits between 2008 and 2011. The number of women attending antenatal care increased from 325 in 2008 to 444 in 2011. Figure 2 depicts the age distribution of pregnant women indicating a high number of pregnancies in adolescent women. The median age of pregnant women attending antenatal care was 23 years. Frequency of antenatal care attendance was assessed systematically in 2010. Then, 323, 257, and 234 women attended first, second and third antenatal care visits at the hospital. Most women attended at a gestational age of 17–28 weeks for their first antenatal care visit.

An average of 273 deliveries per year was recorded at the departmental hospital in the period from 2008 to 2010. Due to a lack of capacity to perform Caesarean sections at this hospital all pregnant women requiring surgical intervention were evacuated to the Albert Schweitzer Hospital in Lambaréné located approximately 80 km North from Fougamou. Among all deliveries in the department of Tsamba-Magotsi an estimated 10% (58/552) occurred at home. The proportion of premature deliveries as defined by gestation below 37 weeks was 7% (42/571). Prevalence of low birth weight defined as less than 2,500 g was 13% (71/555). In 2008 the stillbirth rate was 2% and 7% (95% CI 4.7–10.2%) of 379 women reported at least one prior stillbirth.

Between 2008 and 2011 peripheral blood of 1,646 pregnant women was screened microscopically for *Plasmodium* infection at first presentation to antenatal care. Intermittent preventive treatment of malaria in pregnancy with two doses of sulfadoxine-pyrimethamine was provided to all pregnant women during the study period. At first presentation for antenatal care 13–19% were microscopically positive for *Plasmodium* infection.

Serological screening for infectious diseases was performed for all pregnant women presenting for antenatal care. The prevalence of HIV, HBV and *Treponema pallidum* infection was 6%, 7%, and 3%, respectively (Table 1). Four of the 33 HIV positive women were co-infected with hepatitis B virus and one patient was co-infected with HIV and *T. pallidum*.

**Table 1:** Distribution of infectious diseases in pregnant women 2010–2011(no malaria)

Pathogen	Number of cases	Positive cases	%	95% CI	Method
HIV	550	34	6.2	4.2–8.2	rapid test
HBV	506	37	7.3	5.0–9.6	rapid test
T. pallidum	518	13	2.5	1.1–3.4	rapid test
S. haematobium	591	40	6.8	4.9–9.1	Urine microscopy
Microfilaria	144	32	22.2	15.3–29.1	blood smear
Microfilaria	56	25	44.6	31.3–58.5	leuco-concentration

The prevalence of *S. haematobium* egg excretion in urine at first antenatal care visit was 7% (95% CI 5–9%; Table 1) in 591 HIV negative pregnant women. One hundred forty-four thick blood smears were investigated systematically for the presence of microfilariae in peripheral blood. In 32 samples (22%, 95% CI 15–29%) microfilariae were detected with 11 *M. perstans*, 9 *L. loa*, and one co-infection (no species determination possible in 12 samples). Saponin-concentration was performed for 56 pregnant women and 25 (45%, 95% CI 31–59%) had a positive result (*n* = 14; 25% *M. perstans*; *n =* 7; 12.5% *L. loa*; *n* = 4 no species determination possible).

## Discussion

This study characterizes the disease spectrum and burden of infectious diseases for the rural department Tsamba-Magotsi. Current best estimates of size and structure of the population are based on a governmental census performed in the year 2003 [5]. Since then no updated data are available and no follow-up census has been performed by national institutions. Based on an estimated population growth rate of 1.94% in Gabon and considerable mobility of residents the current population size cannot be reliably determined [12]. However, based on observations and reports of local community representatives, the total population of this department is regarded as constant despite considerable mobility of the young adult population. Interestingly, more than a third of the department population resides in the town Fougamou. The population structure indicates high birth rates and is indicative of an expansive population pyramid.

The collation of epidemiological data from hospital registries and epidemiological surveys conducted in the department Tsamba-Magotsi provides evidence that infectious diseases remain the most important cause for consultation at local health care institutions. Almost three quarters of all health care contacts were due to communicable diseases with malaria being the most frequent condition. Whereas these data indicate that infectious diseases are of high importance, it may also be indicative for the principal services of the health care sector. Arguably, clinicians and laboratories in remote sub-Saharan African regions may be skewed to the management of infectious diseases as opposed to chronic non-communicable diseases. WHO statistics indicate that infectious and parasitic diseases account for 42% of the total burden of diseases in Africa when measured as disability adjusted life years (DALY) [1]. At the same time communicable diseases are responsible for 58% of total DALYs in Gabon [6, 13]. Although these burden of disease measures cannot be directly compared with data from this study, results from this survey demonstrate that the epidemiological transition has hardly begun in this rural region of Gabon.

Malaria epidemiology shows a perennial transmission pattern in this region of Gabon with seasonal variations following pluvial cycles. *Plasmodium* infection rates in the surveyed populations ranged from 14% to 77% with an average of 46% in the 2–10 year olds. These data indicate even higher malaria burden in this rural community than suggested by international estimates of the Malaria Atlas Project (PfPR_2–10_) [7, 14]. Pregnant women showed a prevalence of peripheral parasitaemia at first antenatal care visits ranging between 13 and 19%. Age specific prevalence of *Plasmodium* infection indicates that highest prevalence is observed in children aged 5–18 years. These data are in line with previous results from the provinces of Ogooué Ivindo and Haut-Ogooué indicating no important reduction of malaria transmission over the past decade in rural Gabon [15]. This finding is in contrast to reports from urban regions in Gabon and demonstrates the need for intensified malaria control in remote rural regions [16].

Concordantly, data on the epidemiology of major filarial pathogens demonstrate a high prevalence of infection. Prevalence of filariasis increased with age reflecting a cumulation effect and potentially higher exposure of this population to the vector due to increased agricultural activities [17].

Epidemiological data from antenatal care programs and delivery registries indicate an estimated birth rate of 34 confirming high fertility and an expansive population structure. Despite the availability of only one hospital with modest medical facilities in the department, hospital deliveries accounted for more than 90% of total births. This proportion is high compared to other regions in rural Africa [18]. Birth outcome measures including premature delivery and low birth weight demonstrate comparable birth outcomes to other sub-Saharan African regions. Importantly, hospital records indicate a high proportion of young adolescent pregnancies indicating a need for tailored health programs for adolescents and young pregnant women [19, 20].

Pregnant women have been evaluated as a sentinel population for chronic infections in this study. HIV prevalence in pregnant women was higher in this sexually active population than official national prevalence data for Gabon published by WHO (5.2%). However, a prevalence of 6% is in line with a previous report stating a prevalence of 7% in pregnant women attending the Albert Schweitzer Hospital in the neighbouring province of Moyen-Ogooué in 2008 [17, 21]. Whereas HIV prevalence in pregnant women cannot be used as a direct proxy for population prevalence, these data are currently best estimates for the county of Tsamba-Magotsi. Conversely to HIV the prevalence of *T. pallidum* is surprisingly low (3%) compared to previous prevalence estimates of 8 to 26% from other regions of Gabon [22]. Diagnostic data from pregnant women also indicated a high prevalence of urogenital schistosomiasis and the filarial pathogens *L. loa* and *M. perstans* in the study region. Whereas filariasis has a rather uniform geographical distribution in this study region, urogenital schistosomiasis is characterized by highly variable local rates of transmission depending on the presence of snail infested streams and ponds near the respective communities [23, 24]. A mean prevalence of 7% in pregnant women is therefore indicative of communities with high prevalence and others without transmission.

This first collation of demographic and epidemiological data for the county of Tsamba Magotsi is not without its limitations. Most epidemiological data stem from screening activities of the recently established medical research centre. These surveys were performed over a period of eight years mostly without standardized follow-up surveys and epidemiological trends can therefore not be deducted from these point prevalence studies. Importantly, underlying population estimates are based on a governmental census performed in the year 2003. No follow-up assessments have been performed since then and current population estimates must therefore rely on these historic data. Finally, the characterization of infectious diseases in the sentinel population of pregnant women provides an overview about the scale of transmission of infections but is not entirely representative for the general population.

## Conclusion

The recently established Medical Research Centre operates in an environment of a rural, African population afflicted primarily by the burden of infectious diseases, in particular malaria and neglected tropical diseases. To serve the local population clinical research projects focussing on malaria and other highly endemic communicable diseases will provide important benefit for the local population and may contribute to economic development. This work will help generating follow-up data on the demography and epidemiology of disease burden and will thus help adequately allocating resources for clinical research and governmental health programs in this rural African region. Finally, these data remind us of the important differences in the pace of the epidemiological and demographic transitions that affect sub-Saharan Africa so unevenly.

## Additional file

Additional file 1: Table S1. source, type of data used in this analysis and period collected. (DOCX 13 kb)
