# Supplementary material for: Demography, maternal health and the epidemiology of malaria and other major infectious diseases in the rural department Tsamba-Magotsi, Ngounie Province, in central African Gabon
Source: BMC Public Health. 2017 Jan 28;17:130. doi: 10.1186/s12889-017-4045-x (PMC5273856; doi:10.1186/s12889-017-4045-x)
Supplement: Additional file 1: Table S1. — source, type of data used in this analysis and period collected. (DOCX 13 kb) [file 12889_2017_4045_MOESM1_ESM.docx]

Additional file 1: Table S1: source, type of data used in this analysis and period collected

| Data | Source | Type | Population | Period | Reference  if applicable |
| --- | --- | --- | --- | --- | --- |
| Demographic burden of communicable and non-communicable diseases | Hospital and Lab registries (routine) | Retrospective | General population (excluding pregnant women) | January to December 2010 | NA |
| Malaria seasonality | Hospital registry | Longitunal and retrospective | Adults and children | January to December 2008 |  |
| Malaria  survey | Field work | Cross-sectional | General population (excluding pregnant women) | February to March 2016 | NA |
| Filariasis | Lab registry (routine) | Longitunal and prospective | General population (excluding pregnant women) | 2008-2009 | NA |
| ANC and delivery data | ANC and Lab registries | Longitunal prospective | Pregnant women | 2009-2011 | NA |
| Shistosomiasis and filariasis during pregnancy | screening | Longitunal prospective | Pregnant women | 2009-2011 | Mombo-Ngoma et al.2015, 2016 |

NA: not applicable; ANC: antenatal clinic
